# *Byōseki* and pathography: Their commonalities and differences

**DOI:** 10.1002/pcn5.70102

**Published:** 2025-04-21

**Authors:** Shinnosuke Saito

**Affiliations:** ^1^ Department of Mental Health Jichi Medical University Saitama Medical Center Saitama Japan

**Keywords:** creativity, Joyce Carol Oates, pathography, Paul Julius Möbius, Sigmund Freud

## Abstract

The German psychiatrist Paul Julius Möbius began to use the term *Pathographie* in a new sense: a psychiatric biography of a historical figure that focuses on their pathological aspects*. Byōseki*, which originated from Möbius's *Pathographie*, refers to a uniquely Japanese practice that explores the relationship between creativity and psychopathology. It is also the English translation of the term “pathography,” although the two terms differ significantly in both definition and usage. Originally, “pathography” was defined as “a description of disease,” but it eventually shifted to “a description of an individual or of a community through disease.” In the medical context, it is used mainly in the sense of “medical, psychiatric or psychoanalytic case study of a historical figure” or “patient narratives of illness.” In 1988, a newspaper article redefined pathography as a despicable and ugly biography that emphasizes not only disease but also the negative aspects of life. It can be noted that the above shifts in usage and the addition of new usages in the English term “pathography” were directly or indirectly influenced by Möbius's *Pathographie*. Although the essence of pathography is rooted in “patho‐ (disease, suffering),” the essence of *Byōseki* centers on “creativity.” The two overlap only when referring to medical, psychiatric or psychoanalytic case studies of historical figures. Under each of these terms, a rich body of descriptions of human experience has been archived. Knowing the exact definitions and usages of these terms is crucial for more people to properly access these archives.

## INTRODUCTION

The German word *Pathographie* originally meant “the description of the forms of the disease”^1^ or “description of disease,”^2^ combining the Greek root words *πάθος* (disease) and *γράφω* (describe). It was the German psychiatrist Paul Julius Möbius (1853–1907) who began to use the word in a new sense. At the turn of the century, he published psychiatric biographies of historical figures such as Rousseau (1889), Schopenhauer (1899), Goethe (1898), and Nietzsche (1902). He called these works *Pathographie*.^3^ In the preface to a collection of his works^3^ published in 1903, Möbius argues that in order to understand a great work, it is important to understand the person who created it, that all great people have a pathological side, that ordinary biographers either fail to notice it or turn a blind eye to it, and that psychiatrists are the most suitable people to distinguish between a person's healthy and pathological sides and evaluate them. Throughout the essay, he strongly asserts that psychiatrists have a significant role to play in writing the biographies of great people. Although Möbius never clearly defined the term *Pathographie*, it is generally thought that he used the term to refer to “a psychiatric biography of a historical figure that focuses on the pathological aspects.”^4^, ^5^, ^6^

In the early 1900s, Möbius's *Pathographie* was introduced to the English‐speaking world and, as it underwent various modifications, it influenced the English term “pathography.”

In 1909, *Pathographie* was also introduced to Japan when the medical historian Fujikawa^7^ translated the term into Japanese as *Byōshi* (病志). Since then, *Pathographie* has been given various Japanese translations, and is today known as *Byōseki* (病跡).

Although *Byōseki* originated from Möbius's *Pathographie*, it has developed in a unique way, with an emphasis on “creativity,” and is now a uniquely Japanese practice that explores the relationship between creativity and psychopathology. Tanaka^8^ defined it as “a medical anthropological approach that examines the relationship between creation and psychiatric disorders through psychopathological and psychoanalytical lenses using case studies.” As can be inferred from this definition, *Byōseki* is an academic practice carried out mainly by psychiatrists and psychologists. The academic field that discusses *Byōseki* is called *Byōseki‐Gaku* (病跡学), which in Japan is a subfield of psychiatry.

Presently, the Japanese Society of Psychiatry and Neurology officially designates *Byōseki* as the Japanese translation of the German word *Pathographie* and the English word “pathography.”^9^ However, *Byōseki* and pathography, as I discuss below, differ significantly in both definition and usage. When translated as pathography, *Byōseki* will likely be misunderstood and subject to prejudice. For this reason, in this paper I will first explore the definitions, usages, and connotations of the term “pathography” and then compare them with those of *Byōseki* to clarify the commonalities and differences between the two.

## THE TERM “PATHOGRAPHY” IN ENGLISH DICTIONARIES

### The Oxford English Dictionary

First, I will discuss the definition and usage of the term pathography in a representative English dictionary. *The Oxford English Dictionary* (OED)^10^ defines “pathography” as follows:

1. A description of disease. Rare. Now historical. (1848–)

2. The study of the life of an individual or the history of a community with regard to the influence of a particular disease or disorder (as a countable noun) a study or biography of this kind. (1917–)

The first definition is based on the following contribution to the 7th edition (1848) of the medical dictionary *Medical Lexicon* by the physician Robley Dunglison^11^:

Pathography, *Pathographia*, from *πάθος*, “disease,” and *γράφω*, “I describe.” A description of disease. (The underlined part was adopted by the OED.)

The OED also states that this is the first recorded use of the word in English. The example sentence given is a quote from the book *The Man Who Mistook His Wife for a Hat* (1985) by the neurologist Oliver Sacks:

Hippocrates introduced the historical conception of disease, the idea that diseases have a course, from their first intimations to their climax or crisis, and thence to their happy or fatal resolution. Hippocrates thus introduced the case history, a description, or depiction, of the natural history of disease―precisely expressed by the old word “pathography.” Such histories are a form of natural history―but they tell us nothing about the individual and *his* history; they convey nothing of the person and the experience of the person as he faces and struggles to survive his disease. There is no “subject” in a narrow case history; modern case histories allude to the subject in a cursory phrase (“a trisomic albino female of 21”), which could as well apply to a rat as a human being.^12^ (The underlined part is the sentence adopted by the OED; the emphasis is in the original)

Here, Sacks uses the term pathography to refer to “a description or depiction of the natural history of disease,” and he also suggests that the term is used to describe *disease itself*, as something separate from the sick subject (person). He also states that this has been the traditional usage of this term.

If we look at the second definition, which is more recent than the first definition and the one currently in use, we can see that the focus of the description has shifted from *disease itself* to an individual or a community. What brought about this change? The OED cites as the earliest example of this usage a passage from the English translation (originally in German) of *The Psychoanalytic Method* published in 1917 by Oskar Pfister:

The history of the Catholic sainthood affords the analytic pathography an inexhaustible material which substantiates the interesting statement of the experienced Friedrich von Hardenberg (Novalis): “It is strange that the association of voluptuous pleasure, religion and cruelty has not long ago brought to the attention of men the intimate relationship and common tendency of these.” Psychologically considered, this assertion is absolutely correct.^13^ (pp. 573–574. The underlined part is the sentence adopted by the OED.)

Pfister then goes on to reinterpret the religious efforts of Catholic saints in terms of sexual desire. A Swiss Lutheran minister and psychoanalyst, Pfister developed a personal relationship with Freud that remained unclouded to the end of their lives.^14^ What is implied in pathography here can naturally be seen as a Freudian psychoanalytic understanding of the historical figures.

Actually, this is not the first example of this usage. In the English translation of Freud's *Eine Kindheitserinnerung des Leonardo da Vinci* ^15^ published in 1916, the term “pathography” appears at the beginning of the final chapter. Freud discusses pathography here as a biographical study that focuses on the way pathological or neurotic elements in a person's life can illuminate other facets of that life (pp. 130–131). According to Lang,^5^ who details the history of attempts by psychiatrists and psychoanalysts to understand creative genius, there was much discussion at the Vienna Psychoanalytic Society, Freud's home base, about the issues of creativity and genius from around 1906, centering on psychoanalytic pathography,^16^ which applied psychoanalytic methods to Möbius's *Pathographie*. Taking this background into account, it can be thought that Freud's (and Pfister's) “pathography” originates from Möbius's *Pathographie*.

Prior to this, in 1912, the German physician Friderich Kanngiesser published an article in the *Glasgow Medical Journal* entitled “Notes on the pathography of the Julian dynasty.”^17^ The paper lists the physical, psychiatric, and psychological problems of the emperors of the Julian–Claudian dynasty and their relatives, including epilepsy and syphilis in Julius Caesar, the abnormal personality of Augustus, the alcoholism of Claudius, the delusional epilepsy of Caligula, and the alcoholic paranoia of Nero. Here, the term “pathography” is used in the sense of a medical or psychiatric case study of a great historical figure, which also can be regarded as a usage that refers to Möbius's *Pathographie*.

As far as I could confirm in this survey, this is the earliest example, but in any case, it is thought that Möbius's *Pathographie* was brought to the English‐speaking world in the early 1900s, rather indirectly, through translated works such as those of Freud. This may have given the term “pathography” a new meaning, depicting an individual (especially a historical great figure) through their disease, rather than *disease itself*.

### Other dictionaries

A survey of other English‐language dictionaries reveals a unique definition not found in the OED. *The Collins English Dictionary* provides two definitions of “pathography” similar to those of the OED but adds the following definition: “a biography that focuses on the negative aspects of its subject.”^18^ *Merriam‐Webster Dictionary* also provides the following definition of pathography: “a biography that focuses on a person's illnesses, misfortunes, or failures; *also*: sensational or morbid biography.”^19^ *The American Heritage Dictionary* ^20^ also gives the same definition as the two dictionaries above, and in addition, it includes an example sentence by the American novelist Joyce Carol Oates.

On August 28, 1988, the American novelist Joyce Carol Oates introduced pathography as a new sub‐genre of biography in an article she contributed to *The New York Times*.^21^ At first, she simply described it as “hagiography's diminished and often prurient twin” and explained the differences from traditional biography as follows:

By contrast, pathography typically focuses upon a far smaller canvas, sets its standards much lower. Its motifs are dysfunction and disaster, illnesses and pratfalls, failed marriages and failed careers, alcoholism and breakdowns and outrageous conduct. Its scenes are sensational, wallowing in squalor and foolishness. Its dominant images are physical and deflating. Its shrill theme is “failed promise” if not outright “tragedy.”

As an example of pathography, she takes up *Jean Stafford: A Biography* by David Roberts,^22^ the author of four books on the outdoors. She criticizes the book, saying that it depicts the “numbingly repetitive and emetic life of Jean Stafford” and “falls into pathography's technique of emphasizing the sensational underside of its subject's life to the detriment of those more scattered, and less dramatic, periods of accomplishment and well being.”

There were more than a few criticisms of Möbius's *Pathographie*, saying that it undermined the idealized heroes of Germany by exposing their mental illnesses.^3^, ^5^ There is no denying that *Pathographie* could have sullied the honor of the deceased by exposing hidden, sometimes shameful, facts. For this reason, *Byōseki* researchers are required to give careful consideration to the relatives and relationships of the subject, and to adopt a fair and scientific attitude toward the facts.^8^ Looking at Ms. Oates's definition, we can see that her “pathography” can be regarded as a malicious, unconsidered, and unfair amplification of the negative aspects of *Pathographie*. Although it is not clear whether she was aware of Möbius's *Pathographie*, and the details of how she came up with this usage are not known, it can be said that her “pathography” appears to have inherited certain aspects―especially negative aspects―of *Pathographie*.

## “PATHOGRAPHY” IN A MEDICAL CONTEXT

As shown above, the term “pathography” has a broad definition. Next, I would like to survey the medical literature to clarify how it is specifically used.

In PubMed, I searched for the keyword [pathograph*] (forward match), considering the word change of pathography, pathographies, pathographic, pathographer, and so on, and checked the usage in the corresponding article. Because it is not possible to perform backward matching searches in PubMed, a limitation of this survey is that it does not cover terms such as “neuropathography,” for example. A total of 206 articles were included, from which those whose main text was written in a language other than English were excluded. Finally, 110 articles were selected for this survey, and the usage of “pathography” in these articles was examined. If the definition of “pathography” was explicitly stated in the text, the usage was determined accordingly; otherwise, the usage was determined from the main contents of the article. The results are shown in Table 1.

**Table 1:** “Pathography” in PubMed.

Medical, psychiatric, or psychoanalytic case study of a historical figure (43 articles, 39.1%)
Patient narratives of illness (46 articles, 41.8%)
Description of a disease (4 articles, 3.6%)
Clinical data, such as medical history (3 articles, 2.7%)
Paleopathography (2 articles, 1.8%)
Multiple usages (5 articles, 4.5%)
Other (3 articles, 2.7%)
Usage unknown (4 articles, 3.6%)

### Medical, psychiatric, or psychoanalytic case study of a historical figure

About 40% of the articles used “pathography” to mean a medical, psychiatric, or psychoanalytic case study of a historical figure. However, a closer look reveals that the meaning and purpose of pathography vary somewhat from author to author. The following is an explanation by Khalil et al.,^23^ which is almost identical to the definition of *Byōseki*:

A pathography can be defined as ‘the study of the effects of an illness on the writer's (or other artist's) life or art, or the effects of an artist's life and personality development on his creative work.’

However, usage similar to *Byōseki* is rare (the others are Kenyon^24^ and Schioldann^25^), and the understanding of “pathography” in many articles is as follows:

The terms *retrospective diagnosis* and *pathography* are now used interchangeably, although they have other meanings apart from the diagnosis of illnesses in famous persons. The word *pathography* originally referred to retrospective psychiatric diagnosis, but it now includes retrospective medical and psychiatric diagnoses.^26^ (The emphasis is in the original.)

Muramoto^27^ also argues that “pathography” has the meaning of retrospective diagnosis, and that it originates from *Pathographie*, which was introduced by such German neuropsychiatrists as Julius Möbius, Ernst Kretschmer, and Karl Jaspers. In this way, it is generally thought that the term “pathography” is synonymous with *retrospective diagnosis*, and while it originally referred to psychiatric diagnosis, it now includes medical diagnosis as well. This can be seen as an extension of the concept of Möbius's *Pathographie* to the field of physical medicine. Perhaps with this kind of understanding as a background, retrospective diagnoses of historical figures have been reported under the name of pathography, especially those dealing with medical diagnoses, which have been prominent since the latter half of the 1990s. For example, there are reports of neurosyphilis or chronic heavy metal poisoning in Karen Blixen,^28^ cerebrovascular disease in Georg Friedrich Handel,^29^, ^30^, ^31^ temporal lobe epilepsy in Socrates,^32^ Nietzsche's meningioma,^33^ Mozart's postinfectious sequelae^34^ and chronic kidney disease,^35^ porphyria in Christian VII of Denmark,^36^ acute intermittent porphyria in Van Gogh,^37^ and neurosyphilis in Anglophone composers and jazz musicians.^38^

### Patient narratives of illness

In PubMed, the most common usage of “pathography” is to refer to patient narratives of illness. Almost all of these are directly or indirectly based on the work of Anne Hunsaker Hawkins from the 1980s. Hawkins^39^ points out that a genre of books that is uncommon before 1950 and rarely found before 1900 “seems to have emerged *ex nihilo*” (p. 3), that is, books describing personal experiences of illness. In a 1984 paper, she defined “pathography” as “a generic term designating all narrative descriptions of illness” and assigned this name to the genre.^40^

In the book *Reconstructing Illness: Studies in Pathography*,^39^ Hawkins gave a more limited definition of “pathography” as “a form of autobiography or biography that describes personal experiences of illness, treatment, and sometimes death” (p. 1) and further showed that it can be divided into three groups: (1) testimonial pathographies, (2) angry pathographies, and (3) pathographies advocating alternative modes of treatment (p. 4). In the footnotes of this book, she describes in detail her encounter with this term:

I first encountered the term “pathography” when reading Oliver Sacks's *Awakenings*, where the word is used to refer to biographies that combine science and art—“the most perfect examples” of which are “the matchless case‐histories of Freud.” It must be observed that in the 1990 edition of *Awakenings*, Sacks adds a few sentences to a footnote (on the same page) where he again uses the term “pathography,” though this time with rather a different meaning. Here, the term is used pejoratively to refer to case histories that are “histories of disease” rather than “histories of people, histories of life.” (p. 177)

In this way, Hawkins shows that “pathography” was a term that Sacks did not have a fixed opinion on, and then goes on to explain Freud's pathography:

Freud uses the word “pathography” in *Leonardo Da Vinci and a Memory of His Childhood* (the term may well appear in other of his writings as well). Pathography here refers to a biographical study that focuses on the way pathological elements in a person's life can illumine other facets of that life. Thus, Freud calls his study of Leonardo a pathography, observing that “the aim of our work has been to explain the inhibitions in Leonardo's sexual life and in his artistic activity.” I use “pathography” to refer to an autobiographical or biographical narrative about an experience of illness. (p. 178)

Given that Hawkins also stated in a paper published in 1984 that she borrowed the word “pathography” from Freud,^40^ it is likely that she derived her own usage of pathography based on Freud's. It can be thought that Möbius's *Pathographie* was received by Hawkins via Freud and was expanded as a term for patients to tell their own narratives.

### Other usages

Four papers adopt “a description of disease,” an outdated usage.^41^, ^42^, ^43^, ^44^ Three reports use the term “past history or medical history.”^45^, ^46^, ^47^ For example, it is used as follows:

“Three‐no” pathography: with no history of abdominal operation, abdominal trauma, or abdominal infection.^45^

Two reports use the term in the sense of paleopathography, which is the analysis of archaeological remains in pursuit of diagnostic information.^48^, ^49^ Five reports address multiple uses of “pathography” or compare them, as the present article does.^26^, ^50^, ^51^, ^52^, ^53^

## *BYŌSEKI* AND PATHOGRAPHY: THEIR COMMONALITIES AND DIFFERENCES

Until recently, *Byōseki* had never been introduced outside of Japan. Tanaka^8^ is perhaps the first to do so, referring to *Byōseki* as “pathography in Japan” and providing a detailed introduction to its definition, history, and scholarly achievements. The following is a brief description of *Byōseki*, based mainly on his article.

The origins of *Byōseki* can be traced back to philosophical questions about creativity and madness in ancient Greece. It is considered to be based on Möbius's studies, called *Pathographie*, that *Byōseki* was established in its present form as a form of biography that examines mental illness and creativity in relation to the course of a given person's life.^6^ Later, Jaspers defined *Pathographie* as “the term given to biographies which aim at presenting those aspects of psychic life of interest to the psychopathologist and clarifying the significance of these phenomena and the particular events for the creativity of the individuals concerned.”^54^ After World War II, general overviews of *Byōseki* were published in Japan,^6^, ^55^, ^56^, ^57^, ^58^, ^59^, ^60^ and the definitions of *Byōseki* given in those articles basically follow the definition proposed by Jaspers. Given that Tanaka's definition,^3^ which I presented at the beginning of this article, is similar to these, we can see that today, the definition of *Pathographie* by Jaspers, which discusses the relationship between mental illness (psychopathology) and creativity, remains alive and well.

The narrow definition of *Byōseki* is as above, but the scope of the term has been expanding. For example, since 1980, statistical studies on the relationship between creativity and mental illness have attracted attention in English‐speaking countries^61^, ^62^, ^63^, ^64^, ^65^, ^66^; however, in Japan, these studies are treated as *Byōseki* studies because they belong to the lineage of *Sammelpathographie* (collective pathography, 集団病跡学; *Syūdan Byōseki*‐*Gaku*), which is a genetic, genealogical, or statistical study of a large number of cases, such as those of Lombroso, Lange‐Eichbaum, and Kretschmer. Research into the creativity of *art brut* (outsider art) is also a popular topic of *Byōseki* studies.^67^, ^68^, ^69^, ^70^ As another example, Tanaka^3^ introduces salutography as a new field of *Byōseki*. Aaron Antonovsky, a medical sociologist, proposed the concept of salutogenesis to describe an approach that focuses on factors that support human health and well‐being rather than the factors that cause illness (pathogenesis).^71^, ^72^ The prefix “saluto” is derived from the Latin word *salus* (health, well‐being or welfare). Salutography is a Japanese concept that applies the methodology of salutogenesis to *Byōseki*, and it has been the subject of much discussion.^73^, ^74^, ^75^, ^76^, ^77^, ^78^ *Byōseki* has become prominent, especially in recent years, for its attempt to de‐focus the pathological and take a more inclusive view of the creator, and this is an example of such a move. *Byōseki* today is therefore considered to have moved away from Möbius's *Pathographie*, which focuses solely on the pathological aspects. Meanwhile, the term *Byōseki* is occasionally used by Japanese neurologists and others to mean “retrospective medical diagnosis of a historical figure,”^79^, ^80^, ^81^, ^82^, ^83^, ^84^ in the same way as the English term “pathography,” although this usage falls outside its narrow definition.

Looking at the broad extension of *Byōseki* in this way, we can see that the intention of this term is “psychiatric research into creation or creativity.”^85^ In particular, “creativity” seems to have been an essential element in the expansion of this term's usage.

Let us now summarize the definitions and usages of “pathography” that we have seen in the previous sections. It was originally defined as “a description of disease,” but it eventually shifted to “a description of an individual or of a community through disease.” In the medical context, it is used mainly in the sense of “medical, psychiatric or psychoanalytic case study of a historical figure” or “patient narratives of illness.” In 1988, a newspaper article redefined “pathography” as a despicable and ugly biography that emphasizes not only disease but also the negative aspects of life, and this definition has been adopted by leading English dictionaries. It can be noted that the shifts in usage and the addition of new usages for the English term “pathography” described above were directly or indirectly influenced by Möbius's *Pathographie*.

We can see in this overview that the essence of this word is literally “patho‐ (disease, suffering),” and various definitions and usages seem to have developed depending on who wrote it (a medical professional or a patient), who the subject was (a historical figure or patients themselves), and what the purpose was (diagnosis or something else). Also, it is notable in this overview that pathography has no connotation of “creativity” whatsoever. The search for pathography in PubMed conducted in this survey turned up only one statistical study about creativity.^86^ Nassia Ghaemi's *A First‐Rate Madness: Uncovering the Links Between Leadership and Mental Illness*,^87^ which discusses how mental illness affected leadership and creativity in leaders of the political, military, and business fields, is undeniably a *Byōseki* study. However, in his afterword to the book, Ghaemi refers to his approach as “psychological history” (p. 266), and the term “pathography” is nowhere to be found in the book. These facts also suggest that in the English‐speaking world, studies on the relationship between creativity and mental illness are generally not associated with the term “pathography.”

**Figure 1:**
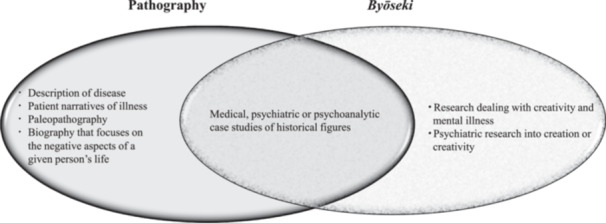
The relationship between “pathography” and *Byōseki* in terms of definitions and usages. Each item included on the left side of the left circle is expressed in Japanese as follows: “description of disease” is *Byōreki* (病歴) or *Shikkan‐no‐Shizenshi* (疾患の自然史), “patient narratives of illness” is *Tōbyōki* (闘病記), “paleopathology” is *Ko‐Byōrigaku* (古病理学), and “biography that focuses on the negative aspects of life” is *Bakuro‐Bon* (暴露本) or *Bakuro‐Kiji* (暴露記事). These are only examples, and several other expressions are recognized. The items included on the right side of the right‐hand circle generally correspond, for example, to themes such as “creativity and psychopathology” in the English‐speaking world. The papers^88^, ^89^, ^90^, ^91^ on this theme deal with issues that are common to *Byōseki*, but the term “pathography” does not appear at all in the papers.

Based on the discussion up to this point, the relationship between “pathography” and *Byōseki* is illustrated in Figure 1. The overlap between the two, although they are referred to by the same word in the English‐speaking world, is quite small. The two overlap only when referring to medical, psychiatric, or psychoanalytic case studies of a historical figures. Pathography has such meanings as “a description of disease,” “patient narratives of illness,” “paleopathology,” and “biography that focuses on the negative aspects of life,” but these are not usually included in *Byōseki*. In contrast, “research dealing with creativity and mental illness” and “psychiatric research into creation or creativity” are central themes pursued by the latter, whereas the former has no such connotations. Considering these differences, misunderstandings and prejudices can emerge in many ways. For example, let us consider the case of a person who was highly praised or disgraced and had many scandalous episodes in their personal life. Even if you wrote a *Byōseki* that focused on their creativity and dealt with the facts in a scientific and neutral manner, it may be perceived, before it is even read, as a despicable and defamatory biography when translated into English as pathography. Or, if the person had an illness, it may be seen as an autobiography describing their experiences. Therefore, I recommend that when being discussed in English, *Byōseki* should be used in its original Japanese form, or with an English explanation added, such as “*Byōseki* (pathography in Japan)” rather than being translated as “pathography.”

The reason this difference arose is thought to be that *Byōseki* has continued to be used as an academic term, whereas “pathography” has been used not only in specific academic fields but also in nonacademic settings. The term *Byōseki* has maintained a relatively stable definition within academic fields, and the term itself continues to be used as value‐neutral, but of course that does not mean there is no criticism against the academic field, namely *Byōseki*‐*Gaku*. On the other hand, “pathography” has been arbitrarily used and valued as an everyday word in various places and settings, and thus may have much broader definitions, usages, and connotations compared with *Byōseki*.

Under each of these terms, a rich body of descriptions of human experience, albeit of different types, has been archived. Whatever the terms may be, these are still the precious heritage of humanity. However, as indicated in this article, the term “pathography” is generally used in ways unrelated to creativity and, in some contexts, it is used to convey a malicious sense. *Byōseki* is at risk of being mistaken in much the same way. Knowing the exact definitions and usages of these terms is crucial for more people to properly access these archives.

## AUTHOR CONTRIBUTIONS

Shinnosuke Saito is the only author of the manuscript.

## CONFLICT OF INTEREST STATEMENT

The author declares no conflicts of interest.

## ETHICS APPROVAL STATEMENT

N/A.

## PATIENT CONSENT STATEMENT

N/A.

## CLINICAL TRIAL REGISTRATION

N/A.

## Data Availability

N/A.
